# Identifying, categorising, and mapping actors involved in resilience in healthcare: a qualitative stakeholder analysis

**DOI:** 10.1186/s12913-024-10654-4

**Published:** 2024-02-22

**Authors:** Veslemøy Guise, Mary Chambers, Hilda Bø Lyng, Cecilie Haraldseid-Driftland, Lene Schibevaag, Birte Fagerdal, Heidi Dombestein, Eline Ree, Siri Wiig

**Affiliations:** 1 SHARE - Centre for Resilience in Healthcare, Faculty of Health Sciences, University of Stavanger, N-4036 Stavanger, Norway; 2 Kingston University & St. George’s University of London, London, UK

**Keywords:** Resilience, Resilient healthcare, Quality of care, Stakeholder involvement, Stakeholder analysis

## Abstract

**Background:**

Resilience in healthcare is the capacity to adapt to challenges and changes to maintain high-quality care across system levels. While healthcare system stakeholders such as patients, informal carers, healthcare professionals and service managers have all come to be acknowledged as important co-creators of resilient healthcare, our knowledge and understanding of who, how, and in which contexts different stakeholders come to facilitate and support resilience is still lacking. This study addresses gaps in the research by conducting a stakeholder analysis to identify and categorise the stakeholders that are key to facilitating and sustaining resilience in healthcare, and to investigate stakeholder relationships relevant for the enactment of resilient healthcare systems.

**Methods:**

The stakeholder analysis was conducted using a sample of 19 empirical research projects. A narrative summary was written for 14 of the projects, based on publicly available material. In addition, 16 individual interviews were undertaken with researchers from the same sample of 19 projects. The 16 interview transcripts and 14 narratives made up the data material of the study. Application of stakeholder analysis methods was done in three steps: a) identification of stakeholders; b) differentiation and categorisation of stakeholders using an interest/influence grid; and c) investigation and mapping of stakeholder relationships using an actor-linkage matrix.

**Results:**

Identified stakeholders were Patients, Family Carers, Healthcare Professionals, Ward/Unit Managers, Service or Case Managers, Regulatory Investigators, Policy Makers, and Other Service Providers. All identified stakeholders were categorised as either ‘Subjects’, ‘Players’, or ‘Context Setters’ according to their level of interest in and influence on resilient healthcare. Stakeholder relationships were mapped according to the degree and type of contact between the various groups of stakeholders involved in facilitating resilient healthcare, ranging from ‘Not linked’ to ‘Fully linked’.

**Conclusion:**

Family carers and healthcare professionals were found to be the most active groups of stakeholders in the enactment of healthcare system resilience. Patients, managers, and policy makers also contribute to resilience to various degrees. Relationships between stakeholder groups are largely characterised by communication and coordination, in addition to formal collaborations where diverse actors work together to achieve common goals.

**Supplementary Information:**

The online version contains supplementary material available at 10.1186/s12913-024-10654-4.

## Background

Resilience in healthcare is defined as ‘the capacity to adapt to challenges and changes at different system levels, to maintain high-quality care’ [1]. Within a resilience perspective, healthcare is construed as a complex adaptive system characterised by highly dynamic and changing conditions [2]. In such a system, multiple individual yet interconnected stakeholders across all three system levels (micro, meso and macro) deal with emergent variations, disruptions, and uncertainties by facilitating and enacting both short term and long term adaptations in efforts to sustain the quality and safety of the system [3]. The adaptive capacity of a system can therefore be said to be founded in the knowledge, skills, activities, and experiences of the people in the system [4, 5]. Moreover, it may be impacted and influenced by relationships [6–10] and social interactions in response to local circumstances in practice [11, 12]. While healthcare system stakeholders such as healthcare professionals and service managers, patients and their informal carers have all come to be acknowledged as important co-creators of resilient healthcare [13–21], our understanding of who, how, and in which contexts key healthcare stakeholders come to facilitate and support resilience is still lacking and more research is therefore needed [5].

### Stakeholders in resilient healthcare

In general, stakeholders can be defined as persons or groups that can claim ownership, rights or interests in the past, present, and future activities, resources and outputs of an organisation or system, or who are affected by those activities, resources and outputs [22, 23]. Furthermore, stakeholders can be categorised by how they relate to or interact with and within an organisation or system [24]. A *healthcare* stakeholder is any person, group or organisation who provides, receives, manages, regulates, or pays for healthcare, and may include patients, family or informal carers, healthcare professionals, managers, regulatory bodies, non-governmental organisations (NGOs), municipalities, and regional health authorities [25].

Stakeholder analysis has been described as an approach or analytical process used to generate knowledge about actors (individuals, groups, or organisations) of relevance to a particular social or natural phenomenon, policy or process [26–28]. Conducting a stakeholder analysis can enable understanding of a range of stakeholder attributes, such as their positions, roles, behaviours, intentions, interrelations, agendas, influence or power, and interests [28, 29]. It can also help identify different stakeholders’ involvement in and effect on decision-making or implementation processes and can be used to assess the influence and resources they may exert over such processes [26–28]. Information from a stakeholder analysis can for example be used to understand organisational context and to facilitate implementation of new projects, policies, and objectives in an organisation, including service development efforts and quality improvement initiatives, as well as to develop strategies for engaging and managing stakeholders of importance to specific decisions or organisational objectives [24, 26, 27, 29]. Furthermore, conducting a stakeholder analysis is seen as valuable when seeking a collaborative approach to the planning, development and delivery of healthcare services and healthcare innovations [30].

Stakeholder analysis methods have not been widely used to identify and categorise stakeholders relevant to resilience in healthcare, or to investigate relationships between the stakeholders enacting system resilience. A handful of previous resilient healthcare studies have however employed a social network analysis (SNA) approach to analyse and map the roles, positions and relationships among crucial stakeholders that contribute to resilient performance in various complex healthcare settings (see e.g. [11, 12, 31–33]). While it has not been utilised as an approach in this study, stakeholder analysis methods such as SNA make it possible to analyse stakeholders’ contributions to resilience based not on their individual properties but on their interactions with other system actors, with previous findings indicating that different stakeholders influence a system’s resilience in different ways and to different degrees [31].

However, these previous studies have only looked at the roles, positions and relationships of different groups of healthcare professionals relative to adaptations and resilience, whereas the roles, positions and relationships of other key healthcare stakeholders such as patients, family carers, managers, and regulators have not been explored. Therefore, in order to further increase our understanding of *who* the different stakeholders that contribute to resilience are, *how* they are involved in creating and sustaining resilience, and in *what context* different groups of healthcare stakeholders contribute to resilient healthcare, research is needed to identify and categorise all the actors involved in enacting and supporting adaptations that facilitate resilience across the healthcare system, and to map the relationships between them. This study seeks to address these gaps in the research knowledge by conducting a stakeholder analysis to identify healthcare system stakeholders that are key to facilitating resilience in healthcare and investigate stakeholder relationships relevant to enacting resilient healthcare systems. As such, this study is not concerned with the resilience of individuals (i.e., psychological resilience) but rather the contributions that individuals alone or in groups make toward the resilience of the system of which they are a part.

## Aim

The aim of the study was to undertake a stakeholder analysis in a selected sample of empirical healthcare studies to a) identify healthcare system stakeholders that are likely to have an interest or influence on resilience in healthcare, b) categorise those stakeholders that are involved in enacting resilience in healthcare, and c) investigate and map stakeholder relationships of relevance to the enactment of healthcare system resilience. The following three research questions will be addressed:

1. Which healthcare system stakeholders are involved in facilitating resilience in healthcare?
2. How are the different stakeholders involved in adaptations that contribute to resilience in healthcare?
3. What characterises the relationships between stakeholders involved in facilitating resilience in healthcare?

This study will contribute insights into who is involved in enacting healthcare system resilience; the ways in which they are involved; and how and in which circumstances relationships between stakeholders have an impact on the ways they are involved in adaptations that facilitate a resilient system. These are insights that can be used to further develop our knowledge and understanding of how patients and other stakeholders come to facilitate resilient healthcare systems.

## Methods

### Research setting

The stakeholder analysis study described here is a part of the Resilience in Healthcare (RiH) research program [25]. More specifically, it is part of a work package focused on understanding how patients and other key healthcare system stakeholders are involved in creating and sustaining resilient healthcare [5]. The RiH research program is set mostly within the Norwegian healthcare system. Norway has a publicly funded, semi-decentralised healthcare system founded on the principle of equal access to services for all inhabitants regardless of social or economic status and geographical location [34]. The central government is responsible for specialist and ambulatory care, which is delivered by four regional health authorities through 20 hospital trusts. In addition, 356 municipalities of varying geographic and population size are responsible for providing primary care services, including rehabilitation, nursing home care, home care, general practitioner, and after-hours emergency services. The Norwegian parliament functions as the national political decision-making body, while the Ministry of Health and Care Services is responsible for regulation and supervision of the system and for ensuring that services are provided in accordance with national legislation and regulations [34]. The main actors in the Norwegian system are public, though private actors also exist. The private health care sector in Norway is small and well regulated, with private for-profit providers accounting for only around 10% of the total operating cost for somatic services and 13% of the cost of mental health services [34].

### Research design

**Fig. 1:**
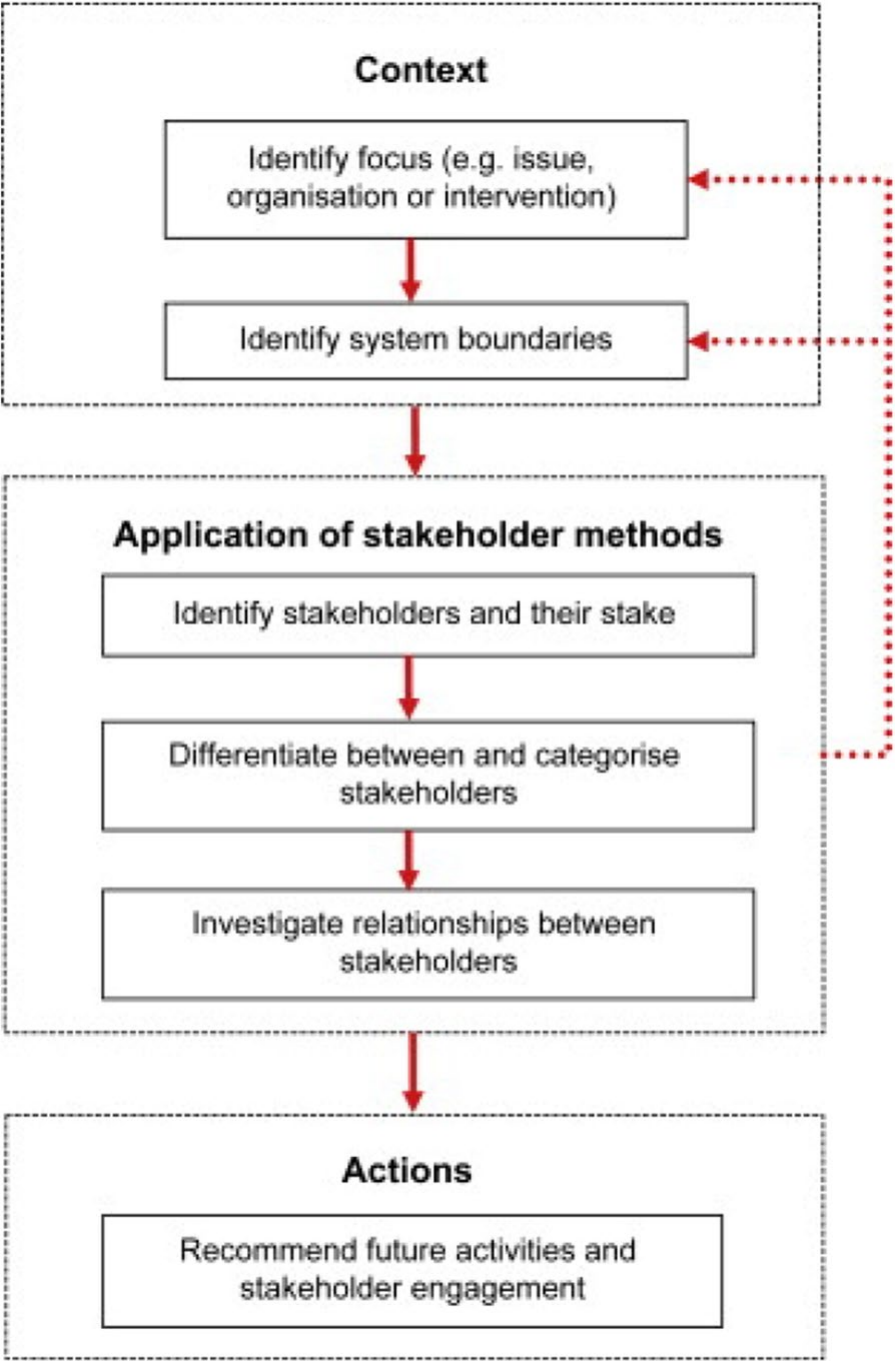
Schematic representation of key methodological steps necessary for stakeholder analysis [28]. Used with permission

The design of the stakeholder analysis was based on the multi-phase process described by Reed and colleagues [28] (see Fig. 1). These are as follows: 1) Definition of the phenomenon of interest and identification of the boundaries for the analysis, which are provided below; 2) Application of stakeholder analysis methods, also described in detail below as part of the Methods section; and 3) Recommendations of future activities and stakeholder engagement, which will be covered as part of the Conclusion of the article.

The first phase of the process described by Reed and colleagues [28] is concerned with clarifying the context of the stakeholder analysis. Before stakeholders can be identified and categorised, we need a clear understanding of the phenomenon under investigation and to establish some analytical boundaries. This is a key part of the process, as how the phenomenon at hand is defined can subsequently affect which stakeholders come to be identified as relevant to said phenomenon. However, while having a clearly defined phenomenon will aid stakeholder identification, who is included or not will likely also depend on the methods used to identify stakeholders, as well as the overall purpose of the stakeholder analysis. From both an ethical and practical perspective, therefore, it is considered sensible to have an inclusive view of stakeholders during the analysis. Once the phenomenon of interest is defined and the boundaries have been clarified, the second phase of the stakeholder analysis process sees the actual application of stakeholder analysis methods. This phase is often comprised of the following three steps: 2a) identification of stakeholders; 2b) differentiation and categorisation of stakeholders; and 2c) investigation of stakeholder relationships in light of the phenomenon of interest. Lastly, the stakeholder analysis process should entail the provision of recommended strategies and processes for effective engagement and involvement of stakeholders in activities such as decision-making processes.

### Defining the phenomenon of interest and the analytical boundaries

The phenomenon of interest in this study was resilience in healthcare, which, as noted, is the capacity to adapt to various challenges and changes in the system to maintain high quality care [1]. In terms of the boundaries of the analysis, adaptive capacity was a central concept as a key feature of resilience in healthcare. The concept of adaptive capacity constitutes adaptations based on reframing, aligning, coping, and innovating in response to diverse demands at different system levels [35]. Adaptations occur in response to challenges, changes and variations in everyday practice and clinical work environments [36, 37] and have been found to take the form of, for example, system-wide adjustments, workarounds, performance trade-offs, sense-making efforts, and improvisation [36, 38, 39]. As will be further explicated below, the analytical process in this stakeholder analysis is therefore founded on identifying instances and examples in the data material of the types of everyday performance variability, challenges and changes that trigger adaptations to facilitate high quality care across different system levels, as well as the stakeholders that are involved when performance variability and adaptations occur.

### Data collection

The data collection process began by screening 50 former and ongoing empirical healthcare services research projects conducted by researchers at SHARE - Centre for Resilience in Healthcare in Norway between 2010 and 2021. SHARE is Norway’s leading research centre related to patient safety, quality and resilience in healthcare and conducts research within a range of healthcare settings featuring stakeholders across all levels of the healthcare system. The screening of eligible projects was done according to a set protocol which included the use of a Quality and Resilience Trigger Tool, which has been published as part of the RiH research programme protocol [25]. The aims of the initial stepwise screening process were to 1) establish which healthcare quality dimensions (e.g., patient safety, continuity of care, patient-centeredness, clinical effectiveness) the projects covered, and 2) if and how resilience was relevant to the quality dimension(s) established. Projects had to be marked for one or more of the four quality dimensions in order to move to step 2. During step 2, resilience was conceptualised as related to actions, activities, and processes, as well as adaptive capacity at individual, team/unit, organisational, and/or system level.

This initial screening process was carried out independently by two research assistants without any prior relationship to any of the 50 projects subject to the screening. During screening, all projects were ranked as dark green (definitive inclusions), light green (highly likely inclusions), orange (possible inclusions; more information needed) or red (not relevant for inclusion). All orange projects, as well as projects where the two research assistants diverged in their initial assessments, were subject to a further independent screening step conducted by a third researcher who used additional publicly available information or approved project plans and/or protocols accessed from project researchers. Any projects still marked orange at this stage, alongside all green or light green projects were subject to consultation with the RiH research team to establish consensus for a final inclusion of projects reflecting a comprehensive range of healthcare settings, quality dimensions, and adaptive capacity at all levels of the healthcare system [25]. This entire process included multiple team discussions on the potential pitfalls of conducting research on researcher colleagues, alongside some principles for how to mitigate these risks with the aim of maintaining the trustworthiness of the RiH project and its associated studies [40].

Of the 50 empirical projects that were screened, 19 were included in the RiH study sample. These 19 studies are presented in Table 1, with details of their relevance to healthcare quality and resilience. At the time of data collection between March 2020 and January 2021, 11 of the projects had been finalised (between 2013 and 2020) while eight were ongoing. The 19 projects together cover a range of research topics and methods and reflect a broad range of empirical settings and study participants. Empirical settings covered in the sample were primary care settings including nursing homes and home healthcare services (projects 3, 5, 12, 14, 17, 18). Five projects were from the hospital setting including surgical (project 6), cancer (project 9), mental health (projects 8, 15), and maternity services (project 1). Another five projects (4, 7, 10, 11, 16) were from transitional care settings and concerned transitions between hospital and primary care services. The remaining projects were from various regulatory settings (projects 2, 13, 19). Research study participants in the included projects encompass a range of different healthcare professionals from both primary and secondary care services, healthcare managers across different system levels, patients, family carers, and regulatory investigators. These are noted in more detail in Table 1.

**Table 1:** Overview of study sample for stakeholder analysis

**No**	**Project status**^a^	**System level**	**Empirical setting**	**Research study participants**	**Relevance to Quality**^b^	**Relevance to Resilience**^b^	**Data sources**
**1**	Finalised 2013	Micro, meso, macro	Hospital maternity services	Midwives, Nurses, Doctors, Ward/Unit managers, Clinical directors, Medical directors, Clinic lead	Patient safety; risk assessment. Patient experiences; patient centeredness.	Implementation of quality improvement measures. Organisational capacity for adaptation; resources, organisation. Larger system capacity for adaptation; regulation; framework conditions.	Narrative Interview
**2**	Finalised 2019	Micro and macro	National regulatory body County Governor’s office	Family carers, Regulatory investigators	Patient experiences; involvement (next-of-kin). Patient safety; adverse events.	Improvements to regulatory methods. Larger system capacity for adaptation; regulation.	Narrative
**3**	Ongoing	Micro and meso	Nursing homes Home healthcare services	Service directors, Nursing home managers, Home healthcare managers, Nurses, Nursing assistants, Enrolled nurses	Patient experiences; involvement.	Development and test of quality improvement intervention. Individual capacity for adaptation; knowledge, competence. Team/unit capacity for adaptation: learning. Organisational capacity for adaptation; resources, organisation.	Narrative Interview
**4**	Finalised 2016	Micro	Transitional care – hospital admission and discharge	Ambulance workers, Nurses, Doctors, Patients, Family carers	Care coordination; care transitions, collaboration across service providers and care levels. Patient experiences; involvement.	Variation in work practice. Individual capacity for adaptation; knowledge, competence, learning, personal characteristics. Team/unit capacity for adaptation: communication. Organisational capacity for adaptation; organisation, culture, resources.	Narrative Interview
**5**	Finalised 2019	Micro and meso	Home healthcare services	Nurses, Enrolled nurses, Care assistants, Physiotherapists, Occupational Therapists, Social workers, Home healthcare managers, Project managers, Professional development managers	Patient safety; risk awareness, risk assessment.	Changes to healthcare processes; variation in work practice. Individual capacity for adaptation; knowledge, competence, learning, personal characteristics. Team/unit capacity for adaptation: communication, learning. Organisational capacity for adaptation: resources, organisation, culture.	Narrative Interview
**6**	Finalised 2013	Micro	Hospital surgical unit (two surgical departments)	Surgeons, Surgical nurses, Anaesthetists, Nurse anaesthetists, Clinic director, Section manager	Patient safety; adverse events.	Variation in work practice; teamwork. Individual capacity for adaptation; knowledge, competence, learning, personal characteristics. Team/unit capacity for adaptation: communication, collaboration, learning. Organisational capacity for adaptation: resources, organisation, culture.	Narrative
**7**	Finalised 2015	Micro	Transitional care – discharge from hospital to primary care	Nurses, Doctors, Head nurses, Patients, Family carers	Care coordination; care transitions. Patient experiences; involvement.	Variation in work practice. Individual capacity for adaptation; knowledge, competence, learning. Team/unit capacity for adaptation: communication. Organisational capacity for adaptation; resources, culture, organisation.	Narrative Interview
**8**	Finalised 2020	Micro	Psychiatric in-patient care	Patients, Nurses, Psychologists, Doctors	Patient experiences; involvement, patient centeredness. Patient safety; risk assessment.	Variation in work practice; stakeholder actions and contributions. Individual capacity for adaptation; knowledge, competence, personal characteristics.	Narrative Interview
**9**	Ongoing	Micro	Cancer department in two hospitals	Family carers, Nurses, Doctors, Ward/Unit managers	Care coordination: collaboration across service providers and care levels. Patient experiences; involvement (next-of-kin).	Stakeholder actions and contributions; knowledge-brokering; co-creation. Team/unit capacity for adaptation; collaboration, learning.	Narrative Interview
**10**	Finalised 2020	Micro and meso	The interface between primary and secondary care – hospitals, nursing homes	Hospital doctors, General practitioners, Nursing home doctors, Nurses, Nursing home managers	Care coordination; collaboration across service providers and care levels, care transitions.	Variation in work practice. Individual capacity for adaptation; competence. Team/unit capacity for adaptation: communication, collaboration.	Narrative Interview
**11**	Finalised 2020	Micro and macro	Hospital and general practice	General practitioners, Radiologists, Trainee radiologists, Radiographers, Doctor case managers	Care coordination; patient pathways, collaboration across service providers and care levels.	Variation in work practice. Individual capacity for adaptation: knowledge, competence, personal characteristics. Organisational capacity for adaptation; organisation, culture. Larger system capacity for adaptation; regulation, framework conditions.	Narrative Interview
**12**	Ongoing	Micro and meso	Municipal home healthcare services	Nurses, Occupational therapists, Older patients / people, Professional development managers	Patient safety; risk awareness, risk assessment.	Changes to healthcare processes; variation in work practice. Individual capacity for adaptation; knowledge, competence, learning, personal characteristics. Team/unit capacity for adaptation; communication, learning. Organisational capacity for adaptation; resources, organisation, culture.	Narrative Interview
**13**	Ongoing	Macro	Employees at the ministry, the directorate, and the Norwegian board of health supervision	Actors at the macro level at governmental regulatory bodies	Patient safety; risk regulation, risk management process, adverse events.	Improvements to regulatory methods. Individual capacity for adaptation; knowledge. Larger system capacity for adaptation; regulation.	Narrative
**14**	Finalised 2020	Micro and meso	Nursing homes	Nurses, Nursing assistants, Care assistants, Nursing home managers, Nursing home doctors	Patient safety; adverse events. Care coordination; collaboration across service providers.	Variation in work practice; trade-offs; disruption. Individual capacity for adaptation; knowledge, competence. Team/unit capacity for adaptation; communication. Organisational capacity for adaptation; resources, organisation, culture.	Narrative Interview
**15**	Ongoing	Micro	Adolescent mental health services	Patients, Family carers, Nurses, Ward/Unit managers	Patient experiences: involvement, patient centeredness.	Variation in work practice; stakeholder actions; co-creation. Individual capacity for adaptation; knowledge, competence, personal characteristics. Team/unit capacity for adaptation; communication, collaboration. Organisational capacity for adaptation; resources, organisation, culture.	Interview
**16**	Ongoing	Micro	Transitional care – discharge from hospital to primary care	Patients, Family carers, Nurses, Doctors, Clinical nutritionists, Physiotherapists, Patient organisations	Care coordination; care transitions, collaboration across service providers and care levels. Patient experiences; involvement, patient centeredness.	Variation in work practice; stakeholder actions; knowledge-brokering; co-creation. Individual capacity for adaptation; knowledge, competence, personal characteristics. Team/unit capacity for adaptation; communication, collaboration. Organisational capacity for adaptation; resources, organisation.	Interview
**17**	Ongoing	Micro and meso	Home healthcare services	Patients, Family carers, Nurses, Occupational therapists, Physiotherapists, Home healthcare managers, Service managers, Case managers	Patient experiences; involvement, patient centeredness.	Variation in work practice; stakeholder actions; co-creation. Individual capacity for adaptation; knowledge, competence, personal characteristics. Team/unit capacity for adaptation; communication. Organisational capacity for adaptation; resources, organisation, culture.	Interview
**18**	Ongoing	Micro	Staff development in home healthcare services	Nurses, Healthcare assistants, Professional development managers, Home healthcare managers	Patient safety; risk assessment.	Variation in work practice; development and test of quality improvement intervention. Individual capacity for adaptation; knowledge, competence, personal characteristics. Team/unit capacity for adaptation; communication, collaboration, learning. Organisational capacity for adaptation; resources, organisation, culture.	Interview
**19**	Finalised 2020	Meso and macro	External assessment prior to ISO certification	External assessors, Hospital managers, Ward/Unit managers	Patient safety, risk management. Clinical effectiveness: effects	Quality improvement measures; organisational development. Organisational capacity for adaptation; resources, organisation, culture. Larger system capacity for adaptation; regulation, framework conditions.	Interview

^a^Project status per time of data collection between March 2020 and January 2021

^b^Based on assessment using the Quality and Resilience Trigger Tool

A narrative summary of between four and seven pages was written for 14 of the 19 projects, which were based on published material from each respective project, such as peer-reviewed articles and completed doctoral theses. At the time of data collection, the remaining five projects did not yet have any publicly available publications to be written into a narrative. All 14 narratives were written by pairs of researchers in accordance with a predefined template for the development of resilience narratives, based on prior theoretical work on the phenomena of resilience in the RiH research program [1, 41]. To this end, each narrative was developed in accordance with four key questions deemed central to understand and operationalise resilience in healthcare:

1. Resilience *for* what: what are the goals, objectives, or activities that resilience is supporting?
2. Resilience *to* what: what is it that triggers, activates, or necessitates resilience?
3. Resilience *of* what: what are the materials and resources that underpin or facilitate resilience?
4. Resilience *through* what: what are the mechanisms, activities or interactions that enact resilience?

In addition, the narratives included details on the respective healthcare settings, system levels, contextual conditions, and stakeholders involved for each project.

Data collection in the stakeholder analysis also entailed 16 individual interviews with researchers from the same sample of 19 projects as described above. One researcher from each of the 19 projects was invited to participate in an interview concerned with gathering data about the specific project they had been involved in. All but three of the 19 invited researchers consented to participate, resulting in 16 interviews. Undertaking research with fellow researcher peers and colleagues about their own research projects is regarded as insider research and may pose several methodological and ethical challenges during recruitment, data collection and analysis processes [42, 43]. The potential risks of this approach were therefore mitigated by adopting several transparent procedures and practices, including the use of a common interview guide, and through the establishment of a formal set of principles for how to handle potential dilemmas when recruiting and interviewing colleagues [40]. These included sending formal recruitment requests with information letters and consent forms via email and involving several research team members in data collection in order to avoid interviews between people with existing friendships or conflicting formal roles, such as an ongoing or previous supervisor/ supervisee relationship between the researcher and the participant.

The interviews explored respective project findings in light of resilient healthcare and touched on a range of topics related to patient and stakeholder involvement in resilience in healthcare, including who, how and in which situations patients and stakeholders are involved in and contribute to resilient healthcare; types of involvement; drivers and barriers to involvement; and contextual factors surrounding patients’ and other stakeholders’ contributions to resilience in healthcare. The interview guide (translated from the original Norwegian) has been provided as a supplementary file (see Additional file 1). Interviews were transcribed verbatim. The 16 interview transcripts, alongside the 14 narratives, made up the data material of the study. As is indicated in Table 1, a majority of 11 projects are represented by both a narrative and an interview, whereas three projects are represented by a narrative only, and five projects are represented by an interview only. Having a combination of narratives and interview transcripts from a majority of the included projects allowed for a deeper exploration and understanding of performance variability and adaptations in the empirical settings represented in these projects. As the interviews were conducted after the narrative summaries had been produced, the interview data functioned to confirm and validate the credibility of the information contained in the narratives, which is one of the strengths of such methodological triangulation [44].

### Data analysis

The application of stakeholder analysis methods was undertaken by a team of healthcare services researchers and followed the three steps outlined by Reed and colleagues [28]: a) Stakeholder identification; b) Stakeholder categorisation; and c) Analysis of stakeholder relationships. How each of these steps was carried out is explained below.

### Stakeholder identification

The first step in the application of stakeholder analysis methods was stakeholder identification, where we applied a deductive-inductive approach to the analysis of the combined data set consisting of interview transcripts and narratives developed from the 19 empirical projects. Data coding in this first step was guided by Research Question 1, focusing on identifying which healthcare system stakeholders are involved in facilitating resilience in healthcare. The initial coding process here was similar to that described elsewhere in the RiH research program [45]. It entailed a close reading of all the narratives and interview texts and was concerned with deductively identifying and coding factors, situations and variations in everyday practice and clinical work environments that trigger performance variability. We furthermore identified and coded for activities and practices that exemplify instances of the performance adjustments or adaptations that were enacted in response to the identified variabilities and triggering factors. Finally, we inductively coded the stakeholders that were involved in the identified factors, situations or variations that triggered the enactments of adjustments or adaptations as expressions of resilience. See Table 2 for an example of how the coding during data analysis was done, illustrated with a data extract each from a narrative and an interview. Identified stakeholders from each of the 19 projects are listed and displayed in Table 3 later.

**Table 2:** Examples of coding process during analysis of narrative and interview data

**Meaning units from narrative (Project 7) that indicate performance variability**	**Factors influencing performance variability**	**Adaptations made in response to variation**	**Stakeholders involved in adaptations**	**Stakeholder relationships**
*Considerable variations were identified in the time (hour of day) the patient was determined medically fit for discharge; discharge process was found to be more rushed when the patients were declared medically fit after noon. This was because of the reduced possibility to prepare the discharge requirements for care transfer if the transfer was to take place the same day. The healthcare personnel stated that time pressure affected precision in their work. When decisions are made later in the day, this also creates time pressure for local municipality personnel who have to initiate processes related to care planning and post-care transfer on their end. This time pressure was exacerbated by financial penalties for delayed discharge; these encourage municipal staff to rush care planning to avoid paying the daily fee*. *The next of kin played an important role as advocates in the decision making regarding post-discharge arrangements. In some cases, the next of kin questioned whether their involvement and persistence had an impact on the level of post-discharge care offered.*	Patient characteristics Degree of familiarity with patient Time of discharge Time pressure to complete task Competing task demands Availability of information Availability of resources External financial demands	Involvement of patients’ next-of-kin in decision-making (at discharge). Family members actively advocate for patients.	Patients Family members Healthcare professionals	Patients and family: fully linked Family and healthcare professionals: collaboration Healthcare professionals from different provider organisations: communication and collaboration
**Meaning units from interview (Project 15) that indicate performance variability**	**Factors influencing performance variability**	**Adaptations made in response to variation**	**Stakeholders involved in adaptations**	**Stakeholder relationships**
*[Patient participation and shared decision-making] is easier to achieve in practice with those [patients] who are motivated and have resources behind them and within their network, then it is much easier to succeed. … For those who seek treatment and believe in it, who are motivated and want it, it is easy to make it happen. For those [patients] who have a more complex picture and who are more unwell, and perhaps have less support, et cetera, then it is far more demanding and … the healthcare personnel and therapists must use their clinical competence and make a discretionary assessment as a provider. You cannot standardise it [patient care] 100%, you have to offer different menus to different people. Some people get a simpler menu, while others get one with many options … or where multiple steps … is what is required to be able to establish collaboration or create a secure environment [for the patient]. Whereas if you were to do that with every single young person who comes into mental health care, then you would not have managed to help more than the tip of the iceberg of those who need it. So an assessment is required along the way by the staff.* *Family members often have an important role in … the follow-up of measures put in place [for the young person] but will depend on their capacity for cooperation and engagement in follow-up over time.*	Patient characteristics Patient needs Patient capacity Degree of family support Family capacity for involvement Staff / team competency Staff / team experience Staff / team capacity Unit / ward capacity	Extra or special efforts to engage patients by healthcare professionals. Involvement and engagement of family by healthcare professionals. Family involvement in follow-up of care.	Patients Family members Healthcare professionals	Patients and family: fully linked Patients and family: not linked Family and healthcare professionals: collaboration Family and healthcare professionals: not linked

**Table 3:** Stakeholder Identification and Categorisation (adapted from Gilmour & Beilin, 2007)

**Project No**	**Patients**	**Family Carers**	**Healthcare Professionals**				**Ward/Unit** **Managers**	**Organisation Managers**		**Regulatory Investigators**	**Policy Makers**	**Other Service Providers**	**Stakeholders according to system level**				**Stakeholder interest/influence**		
			Doctors	Nurses	Allied	Other		Service Managers	Case Managers				Micro	Meso	Macro	External / cross-level	Subjects	Players	Context-setters
**1**	●		●	●		●	●	●					●	●			**P**	**HC, WM**	**OM**
**2**		●								●			●		●			**F**	
**3**	●		●	●	●		●	●		●	●		●	●	●		**P**	**HC, WM**	**OM, RI, PM**
**4**	●	●	●	●		●							●				**P**	**F, HC**	
**5**				●	●	●	●	●			●		●	●	●			**HC, OM**	**PM**
**6**	●		●	●			●	●			●		●	●	●		**P**	**HC, WM, OM**	**PM**
**7**	●	●	●	●							●		●		●		**P**	**F, HC**	**PM**
**8**	●	●	●	●		●	●				●	●	●		●	●		**P, F, HC, OSP**	**WM, PM**
**9**	●	●	●	●			●					●	●			●	**P**	**F, HC, WM, OSP**	
**10**	●	●	●	●			●	●			●		●	●	●		**P**	**F, HC, WM**	**OM, PM**
**11**	●	●	●			●			●		●	●	●	●	●	●		**P, F, HC, OSP**	**PM**
**12**	●	●		●		●		●				●	●	●		●	**P**	**F, HC, RI**	**OM, PM, OSP**
**13**							●	●		●	●		●	●	●			**WM, OM**	**RI, PM**
**14**	●		●	●	●	●	●				●		●		●		**P**	**HC, WM,**	**PM**
**15**	●	●		●			●		●		●	●	●	●	●	●		**P, F, HC, OSP**	**WM, OM, PM**
**16**	●	●	●	●		●					●	●	●		●	●		**P, F, HC, OSP**	**PM**
**17**	●	●		●	●	●	●	●	●				●	●				**P, F, HC**	**WM, OM**
**18**	●			●	●	●	●	●					●	●			**P**	**HC, WM**	**OM**
**19**							●	●				●	●	●		●			**WM, OM, OSP**

KEY: *P* Patients, *F* Family, *HC* Healthcare Professionals, *WM* Ward Managers, *OM* Organisational Managers, *RI* Regulatory Investigators, *PM* Policy Makers, *OSP* Other Service Providers

### Stakeholder categorisation

**Fig. 2:**
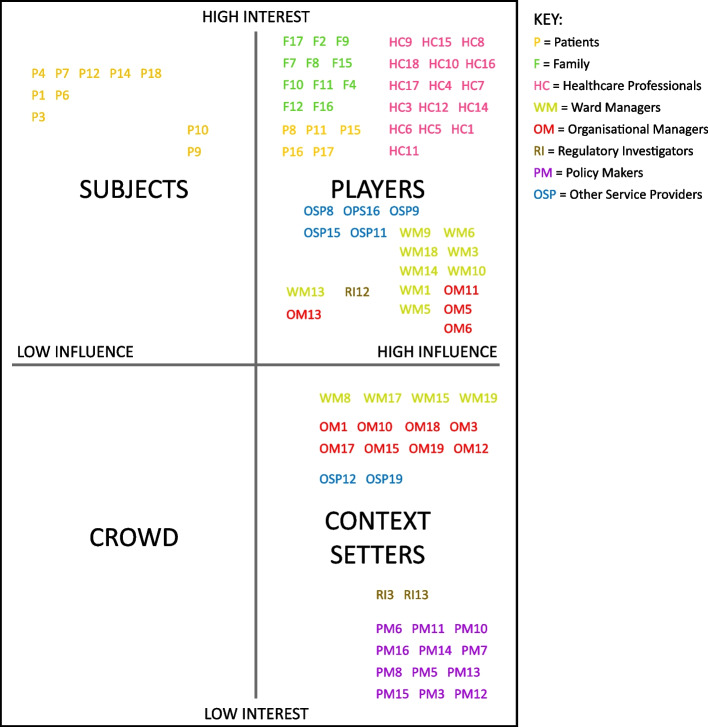
Interest/Influence matrix (adapted from Ackermann & Eden, 2011 [47])

Once all the stakeholders in the combined data set of narratives and interviews had been identified and listed, the second step of the stakeholder analysis was to differentiate and categorise the identified stakeholders. The stakeholders were initially grouped together into broad categories across the full data set [29] to aid subsequent analytical steps [28]. These broad categories were as follows: Patients, Family Carers, Healthcare Professionals, Ward/Unit Managers, Service or Case Managers, Regulatory Investigators, Policy Makers, and Other Service Providers. Next, the stakeholders were categorised according to the system level within which they were found to act, i.e., according to whether they are micro, meso, macro, or external and cross-level actors (see Table 3). Lastly, the final stage of the stakeholder categorisation was done by assessing their level of interest in and influence on performance variability and adaptations that contribute to resilient healthcare (see both Table 3 and Fig. 2). According to previous research, interest and influence are the stakeholder attributes most frequently assessed in stakeholder analyses [46], while influence and interest maps are the tool most often used to aid categorisation [29]. As such, an interest/influence grid was chosen to assess and map the stakeholders in our study, where the stakeholders were positioned according to their level of interest and influence as either ‘Subjects’, ‘Players’, ‘Crowd’, or ‘Context Setters’ [47], as displayed in Fig. 2. To differentiate the stakeholder groups in the figure, they have been marked in different colours, and all identified stakeholders from the 19 projects have been labelled with their respective project number.﻿

‘Subjects’ are stakeholders with medium to high levels of interest but low levels of influence. They have a high stake in the issue concerned but have limited capacity to act to influence outcomes. These stakeholders may however have unrealised influence, which may be activated through alliances with other stakeholders. ‘Players’ are stakeholders with medium to high influence *and* interest and have both the ability to act to influence outcomes *and* the interest to do so. The ‘Context setters’ have medium to high levels of influence but comparatively low levels of interest. They may set the context and define the debate and their position may therefore be critical to the outcome, but they are not active players in the issue to the same extent as other stakeholders. Finally, stakeholders in the ‘Crowd’ have low levels of interest and little or no capacity to influence outcomes. Understanding where stakeholders sit on this grid is central to e.g., developing stakeholder management strategies and ensuring appropriate processes for stakeholder participation.

On this type of grid, then, the influence axis represents the stakeholder’s capacity to act to influence the phenomenon of interest. Influence includes access to and mobilisation of resources and is typically based on a stakeholder’s ability to provide or withhold resources and information, as well as their capacity to exert influence on others and to mobilise other stakeholders and their resources [29, 30]. For the purposes of our study, influence was seen as expressed first and foremost through the level of stakeholders’ *active* involvement in performance variation and adaptations, e.g., through concrete actions taken by the actor or actors involved in identified incidences of performance variation and adaptive capacity. Stakeholders’ level of influence was thus gauged by assessing which stakeholders were *taking action* in the event or process; which stakeholders were *triggering the action* taken by others; and which stakeholders were *facilitating or supporting actions* taken by others. In line with this interpretation, we expect to see some actors perform actions in reaction to certain circumstances, whereas other actors are acted upon or reacted toward by others.

The interest axis illustrates the significance that the issue has to the stakeholder, which can be, for example, personal, political, financial, social, cultural, or a combination of these [29]. Furthermore, the level of interest was gauged by the stakeholders’ proximity to the care events and/or associated work processes described in the data. Proximity was understood both as their physical proximity to a described event or process, e.g., by way of having a *personal* stake in it or not, and as temporal proximity to the event or process in terms of *time and distance*. For our purposes regarding the safety and quality of healthcare, having primarily personal stakes in the issue was thus ranked at a higher level of interest than having political, financial, social or cultural stakes in it. The initial coding using the interest/influence grid was carried out by lead author VG and then discussed with the research team as part of an iterative process during a workshop and subsequent meetings. All team members checked and discussed the placement of all stakeholders from each included project according to their interest in (proximity) and influence on (action-taking) performance variability and adaptations. The purpose of these iterative discussions was to make sure that all the stakeholders were placed in the most meaningful part of the grid according to the events and processes described in the data.

### Stakeholder relationships

Stakeholder relationships in the combined data set of narratives and interviews were analysed using an actor-linkage matrix (see Table 4), which visually maps the links between the key actors central to resilience in healthcare [48, 49]. In an actor-linkage approach, stakeholders, or stakeholder groups, are tabulated in a two-dimensional matrix and the interrelations between them are described using keywords or codes [28]. In practice, all actors are listed down the rows of the table and the same actors are listed across the table columns. The cells in the matrix represent the relationships between stakeholders, and the full matrix shows all interrelations between all the groups of stakeholders [48].

**Table 4:** Actor-linkage matrix (ALM)

	**Patients**	**Family Carers**	**Healthcare Professionals**	**Ward/Unit Managers**	**Organisational Managers**	**Policy Makers**	**Regulatory Investigators**	**Other Service Providers**
**Patients**	Communication Collaboration	Not linked Communication Collaboration	Communication Collaboration	Not linked	Not linked	Not linked	Not linked	Communication
**Family Carers**	Communication Collaboration	Communication	Communication Collaboration	Not linked	Not linked	Not linked	Communication	Communication
**Healthcare professionals**	Communication Collaboration	Communication Collaboration	Communication Coordination Collaboration Fully linked	Fully linked	Coordination Fully linked	Coordination	Communication	Communication Coordination
**Ward/Unit managers**	Not linked	Not linked	Fully linked	Coordination	Communication Coordination Fully linked	Communication Coordination	Communication	Coordination
**Organisational managers**	Not linked	Not linked	Coordination Fully linked	Communication Coordination Fully linked	Communication Coordination	Communication Coordination	Communication	Coordination
**Policy Makers**	Not linked	Not linked	Communication Coordination	Communication Coordination	Communication Coordination	Coordination	Coordination	Coordination
**Regulatory Investigators**	Not linked	Communication	Communication	Communication	Communication	Coordination	Not linked	Not linked
**Other Service Providers**	Communication	Communication	Communication Coordination	Coordination	Coordination	Coordination	Not linked	Not linked

The cells in this matrix represent relationships between stakeholders, indicated by *the degree of contact and collaboration* between the actors noted in the rows and the actors noted in the columns. The degree of contact ranges from ‘Not linked’ to ‘Fully linked’ [44, 45]. The rows contain the stakeholders who are directing the contact and collaboration efforts, such as instances of communication, whereas the columns contain the stakeholders who the contact and collaboration efforts are being directed towards

Key: Not linked (no contact between actors); Communication (actors share information only); Coordination (actors from separate teams or organisations work together to achieve common goals); Collaboration (actors interact as an informal team with specific responsibilities); Fully linked (actors work together as a formal team; mutually plan and share resources to accomplish common goals)

For the purposes of this analysis of the stakeholders involved in adaptations and instances of performance variability that facilitate resilient healthcare, we were interested in determining the degree of contact and collaboration between the identified stakeholders [30], using the following labels adapted from previous work by Harris and colleagues [50] and Schoen and colleagues [51] : Not linked (no contact between actors), Communication (actors share information only), Coordination (actors from separate teams or organisations work together to achieve common goals), Collaboration (actors interact as an informal team with specific responsibilities), and Fully linked (actors work together as a formal team; mutually plan and share resources to accomplish common goals). In this study on healthcare system stakeholders, ‘Collaboration’ was interpreted to include actors who are in a patient-provider relationship, ‘Fully linked’ was taken to mean formal healthcare teams that work together day-to-day according to set plans and goals, for example hospital ward teams or nursing home teams.

## Results

### Stakeholder identification

The stakeholder analysis identified several stakeholders from different healthcare settings and across different system levels who play a role in resilience in healthcare. All healthcare system stakeholders identified as being involved in performance variability and adaptations across the 19 projects are displayed in Table 3.

Patients were identified as stakeholders involved in performance variability and adaptations in 15 projects. Patients feature as stakeholders in five projects from primary care settings (nursing homes and home healthcare services) (3, 12, 14, 17, 18), five projects from transitional care settings (4, 7, 10, 11, 16), and another five projects from various hospital settings, specifically mental health services (8, 15), surgical services (6), maternity services (1), and cancer care services (9). Elderly patients situated in either primary care or transitional care settings feature in a majority of eight projects (3, 4, 7, 10, 12, 14, 17, 18). Of the four projects without patients identified as stakeholders, three (2, 13, 19) were from the regulatory setting and one (5) was concerned with work practice innovation and development in the home healthcare setting.

Family carers were identified as stakeholders involved in performance variability and adaptations in 11 projects. Most of the projects with family as stakeholders are from the transitional care setting (4, 7, 10,11, 16), where informal carers have a key role in supporting and advocating for patients. Three projects concerned hospital settings, specifically mental health services (8, 15) and cancer care services (9), where family also provide considerable support for patients, and two were from the primary care setting (12, 17), where patients living at home are also supported by family. Informal carers were also identified as stakeholders in one project from the regulatory setting (2).

Healthcare professionals were identified as stakeholders involved in performance variability and adaptations in 16 projects. Since healthcare professionals are a large heterogenous group and many projects have a range of different healthcare professionals involved, they have been divided into four subgroups: medical ‘doctors’ (identified in 11 projects), registered ‘nurses’ (identified in 15 projects), ‘allied’ healthcare professionals such as auxiliary nurses or healthcare assistants (identified in five projects), and ‘other’ healthcare professionals, which include e.g. midwifes, ambulance staff, physiotherapists, and radiographers (identified in 10 projects). These subgroups have been indicated in Table 3. Healthcare professionals are absent as stakeholders in only three projects, all of which are from the regulatory setting (2, 13, 19).

A large variety of managers at both the micro level and the meso level of the healthcare system were identified as stakeholders involved in performance variability and adaptations in 15 projects overall. Micro level managers such as ward or unit managers were identified in 13 projects, while meso level managers, including professional development managers, hospital managers, nursing home managers, municipal or other local service or case coordination managers, were identified in 12 projects. Managers are absent as stakeholders in only four projects, three of which are from the transitional care setting (4, 7, 16). In Table 3, managers at the micro level are referred to as Ward/Unit Managers, and managers at the meso level are referred to as Organisation Managers, with further division into the subgroups Service Managers or Case Managers.

Stakeholders representing the macro level of the healthcare system are Regulatory Investigators and Policy Makers. Regulatory Investigators were identified as stakeholders in three projects, two of which are from the regulatory setting (2, 13), as well as in a project (3) from the primary care setting, where regulatory stakeholders are present by way of being responsible for law enforcement and control of the regulatory directives that other actors in the system have to comply with. Policy Makers appear as stakeholders in eleven projects (3, 5-8, 10, 11, 13-16), mainly via the various policy documents, national guidelines, etc. that they produce which other stakeholders then refer to as important for how they perform their work.

Finally, a composite group of various other external and cross-level stakeholders termed Other Service Providers were identified as being involved in performance variability and adaptations in seven projects. This diverse group of stakeholders includes private healthcare providers (11), health technology companies (12), patient or family organisations and support services (8, 9, 16), a national training and rehabilitation centre (16), a private healthcare auditing services (19), and child protection services (15).

### Stakeholder categorisation

All the stakeholders who were identified in the previous step were then differentiated and categorised relative to their level of interest in and influence on performance variability and enactments of adaptive capacity. All stakeholders from each of the 19 projects have been labelled with their respective project number and placed on the grid of an influence/interest matrix, as is depicted in Fig. 2. This stakeholder categorisation is also displayed in Table 3. A majority of stakeholders across the 19 projects were placed in the Players category, representing all stakeholder groups apart from policy makers. All stakeholders categorised as Subjects are patients, whereas stakeholders categorised as Context-setters are managers, regulatory investigators, and policy makers. None of the stakeholders in this study were classified as part of the Crowd, which are those with low levels of interest and little or no capacity to influence outcomes. A more detailed description of how the different groups of stakeholders in the Players, Subjects and Context-setters categories influence performance variability and adaptive capacity is provided in the following.

#### Players

All healthcare professionals (projects 1, 3-12, 14-18) are in the Players category, as are all family carers (projects 2, 4, 7-12, 15-17). Managers at both the micro (projects 1, 3, 5, 6, 9, 10, 13, 14, 18) and meso levels (projects 5, 6, 11, 13) are also in this category. Players are the stakeholders that have both a high ability to influence the performances and adaptations that support resilient healthcare *and* a high interest in doing so.

Healthcare professionals play a crucial role in enacting adaptations to support resilience in healthcare. This is done in numerous ways. A key example, of which there are many instances described in the data material, is that healthcare professionals continually adapt to deal with the myriad uncertainties and emergent situations that characterise their everyday work. This happens through, for example, active problem solving and decision-making, workarounds, improvisation, and targeted planning or anticipatory efforts (projects 5, 6, 8, 10, 11, 14, 16). Another type of adaptation enacted by healthcare professionals is their flexible prioritisation and delegation of tasks and responsibilities according to changing circumstances. For example, in one project (1), healthcare professionals were seen to act as resource coordinators helping to triage patients and allocate staff, often based on continually shifting conditions on their ward or unit. A further example is their engagement in trade-offs between competing demands, such as responding to the individual needs of patients versus following clinical guidelines (projects 8, 15), or subverting rules and guidelines in favour of expressing their own professional judgement or autonomy (projects 11, 14). Healthcare professionals are also involved in various adaptations that work to fill gaps in the system. This includes taking on new or extra tasks and responsibilities in addition to their regular workload (project 3, 10, 12, 17).

Family carers also emerge as stakeholders with an important role in performance variation and adaptations. Family act as advocates and supporters of patients in interactions with healthcare services, particularly for vulnerable patients such as frail elderly or palliative patients. For example, family carers would directly pressure healthcare professionals to influence discharge and transfer processes (projects 4, 7, 10). Furthermore, family carers perform a key support function for mental health patients when, for example, there is a lack trust in healthcare professionals (project 8, 15). Family members also have an important function in filling knowledge and information gaps within and across healthcare providers, settings, and system levels, especially where patients are either absent or too unwell to provide, or receive, the information themselves (projects 2, 4, 7, 8-12, 16). Family also fills gaps in the system by directly covering care tasks for healthcare staff, for example when services are busy or understaffed (projects 4, 9, 17). In addition, family carers take on tasks such as monitoring and observing a patient’s condition and symptoms as well as managing treatment regimens and outcomes, particularly after patients have been discharged to the home (projects 8, 9, 15-17).

Five of the projects had patients who were actively involved in enacting adaptations and thus were classified as Players. An example of active involvement is where patients are seen to fill gaps in the healthcare system by taking on work that would not normally be considered a patient task, such as homecare patients providing ad hoc training to home healthcare staff not familiar with specific care procedures (project 17). Another example of patients filling gaps in the system was patients taking on informal support roles for other patients when formal support from healthcare service providers was lacking (projects 8 and 16). Active involvement from patients was also seen where patients’ direct requests, demands or other input had an impact on healthcare professionals’ adaptations and adjustments of formal practice guidelines, such as referral practices in general practice (project 11), or admission practices (project 15) and clinical assessment practices (project 8) in mental health services.

Managers at both the micro and meso levels enact adaptations to support resilience in different ways. An example is their continual allocation and reallocation of tasks and recourses like personnel and equipment according to changing needs and demands such as unexpected increases in patient admissions (project 1). Managers were also seen to step into clinical work roles when the conditions on the ward/unit demanded it, such as during peak hours or when there were staff shortages (projects 3, 18). In addition to such short-term adaptations, managers at both the micro and meso levels are often in charge of more long-term change processes, such as the implementation of new national guidelines or policy initiatives (projects 3, 5, 11, 12).

Five projects had stakeholders identified as other service providers who were categorised as Players (8, 9, 11, 15, 16). In all five of these projects, other service providers offered patients and/or families either healthcare, social, or other training and support services, in either a local or national setting, that the public healthcare services could not or did not provide. An example of this was coping and support courses and other informal services for cancer patients and their families (projects 9, 16), which provided valuable assistance and information that patients and families could not access as part of formal healthcare services.

#### Subjects

The majority of patients are in the Subjects category (projects 1, 3, 4, 6, 7, 9, 10, 12, 14, 18). They have a high stake in adaptations that support resilient healthcare being enacted, but limited capacity to make these adjustments themselves, particularly relative to stakeholders in the Players category. However, the stakeholders in the Subjects category may, as noted, activate their potential for influence through relationships with stakeholders who are Players. An example of this is where different aspects of a patient’s characteristics including their medical condition and treatment or care needs (e.g., being a high-risk maternity patient, elderly, suicidal, in palliative care, or newly discharged) trigger adjustments and adaptations engaged in by other stakeholders, most notably healthcare professionals (projects 1, 3, 4, 7, 8, 12, 15, 17), and family carers (projects 8, 9, 15, 16, 17).

#### Context setters

Stakeholders in the Context Setters category are first and foremost policy makers (projects 3, 5-8, 10-12, 14, 15, 16), in addition to some managers at both the micro (projects 8, 15, 19) and meso levels (projects 1, 3, 10, 12, 15, 18, 19), regulatory investigators (projects 3, 13) and other service providers (projects, 12, 19). These stakeholders may set a lot of the context of clinical work and define issues around healthcare quality and safety, and their positions may be critical to outcomes in practice, but since they are often more removed from clinical settings and the work therein, they are not directly involved in adaptations to the same extent as the stakeholders in the Players category.

The most common group of stakeholders to set the context and parameters for other stakeholders to act within and react to are policy makers. An example is from a project (7) where, due to the changes imposed by the Norwegian Coordination Reform [51, 52], managers and healthcare staff in hospitals and primary care services had to enact adaptations to practice in order to meet novel policy demands, including flexible discharge practices and interorganisational collaboration in discharge planning. Several other projects (3, 5, 6, 11, 12, 14, 15, 16) also featured examples of various policy documents, guidelines, and checklists triggering performance variation and adaptations in clinical practice. These included management regulations, clinical checklists, cancer treatment guidelines, referral guidelines, medication management guidelines, White Papers on implementation and use of e-health services, and patient involvement policies.

Managers at both micro and meso level also contribute to context-setting. A common example of how managers set the context for other stakeholders to enact adaptations is through active facilitation of learning and competence development among staff by making training opportunities and reflexive spaces available (projects 1, 8, 10, 12). Furthermore, managers typically come to influence the context of practice through their daily efforts to impose and uphold the necessary structural parameters and organisational culture that allow other actors in the clinical setting to engage in adaptive practices that uphold the quality and safety of care, as well as through efforts to positively influence organisational budgets and resource allocation decisions to the benefit of their respective ward/unit or department (project 3, 10, 15, 17, 18).

Lastly in the findings on stakeholder categorisation, other service provider stakeholders were categorised as Context-Setters in two projects, namely health technology companies (project 12) and auditors from a private healthcare auditing company (project 19), whereas a further two projects (3 and 13) had regulatory investigators as Context-Setters. Both the regulatory investigators and the external auditors in these projects were seen to set the context for other stakeholders’ adaptations in similar ways by facilitating risk management and quality improvement efforts, with the ultimate intention of getting both staff and management at the micro and meso levels of the healthcare system to reflect on their own practice and engage in improvement work within their respective organisations.

### Stakeholder relationships

The relationships between all identified stakeholder groups have been analysed and displayed using the actor-linkage matrix in Table 4. The interrelations between stakeholders are labelled with keywords that describe the degree and type of contact between stakeholders involved in adaptations and instances of performance variability that facilitate resilient healthcare: Not linked; Communication; Coordination; Collaboration; and Fully linked. The most closely linked stakeholder groups and the bearing that their relationships have on adaptations are described in the following.

Patients have links to other patients, family, healthcare professionals, and other service providers. In the main, patients had their strongest links to family, where they interact together as an informal team with specific roles and responsibilities (i.e., ‘Collaboration’). Examples of where patients and family act as a collaborative team were found in the projects from the transitional care setting, where family carers are closely allied to patients and actively advocate for them and come to influence care decisions prior to, during and after patients are moved between hospital, primary care services, or their home (projects 4, 7, 10, 16). Another example of when adaptations are based on whether or not patients have family present were seen where patients were more likely to be discharged from hospital to home when they lived together with family member who could help with clinical observations and practical care tasks (project 9, 16). Conversely, there were several examples of how healthcare professionals adapted their decision-making when patients lack the support of family (i.e., ‘Not linked’), like delaying the discharge of elderly patients deemed medically fit for discharge when they do not have relatives available to care for them (projects 4, 7, 10).

There were also some examples of links between patients, which were characterised by both communication, i.e., information exchange, and collaboration, i.e., informal team work. An example was seen in a project (16) where patients came together in informal groups to share information about available resources and support options, thereby filling a gap when such information was lacking from formal healthcare providers. This type of peer-to-peer support was initiated with the express function of being an arena for patients to share information and learn from each other with the goal of facilitating greater coping skills in this group of patients. A similar example was seen in the mental health inpatient setting, where patients came to provide mutual care and support for each other on occasions where they felt this was missing from the ward staff (project 8). Furthermore, various peer-to-peer support groups were seen for both patients and families within the adolescent mental healthcare setting, offering information and support beyond what the healthcare services provide (project 15).

Besides their strong ties to patients, family stakeholders also have significant links to healthcare professionals. This was clearly demonstrated in those instances where family carers and healthcare professionals come together to collaborate in the best interests of patients. Healthcare professionals often depend on collaborative relationships with family carers to uphold the safety and continuity of patient care (projects 9 and 17). For example, family carers provide healthcare professionals with crucial information, insights and experiences about the patient and their care history that may lead professionals to adjust their practice and decision-making accordingly. Conversely, healthcare professionals may encourage patients to bring family members to appointments so that they can help patients remember the information they receive, particularly prior to discharge from the hospital (projects 4, 7, 16). In this way, family carers act as an important buffer between the patient and healthcare professionals, filling a function that cannot be replaced by other stakeholders in the system.

Healthcare professionals have links to all the other types of stakeholders and are thus the stakeholder group that have the most links, ranging from ‘Communication’ to ‘Fully linked’. In addition to the crucial connections to family stakeholders that are described above, healthcare professionals have their closest ties to patients, other healthcare professionals, and managers. Several projects (4, 8, 11, 15) contained examples of how collaborative relations between healthcare professionals and patients impact variations and adaptations in practice, such as healthcare professionals actively involving patients in their own care by, for example, asking patients to describe their health challenges in their own words during medical examinations, whereupon discharge or referral practices were adapted to meet various individual needs and preferences of patients. There were also examples of collaborative links between groups of healthcare professionals and patients, such as instances where nurses stepped in to facilitate communication between doctors and patients during consultations (project 16). In addition, healthcare professionals adapt in different ways to provide training and support for patients and their families. For example, they often act as patient advocates (project 4, 7, 10), or they provide training for patients when new equipment is introduced as part of care routines (project 12).

The analysis goes on to show, perhaps unsurprisingly, that healthcare professionals are most closely linked to the colleagues and immediate managers with whom they work together as a formal team (or teams) that mutually plans its actions and shares resources including staff to accomplish predetermined common goals. As was noted above, healthcare professionals commonly enact adaptations to fill gaps in the system, like taking on extra tasks and responsibilities. Adaptations such as these may be enacted in explicit response to the needs of other healthcare professionals or managers on a team, for example to alleviate overworked colleagues (projects 1, 16, 18) or to compensate for a lack of competence in other team members (project 14). Indeed, healthcare professionals often enact adaptations with the intention of supporting each other in various ways. For example, they may act to bridge information gaps, either by contributing to the transfer of information between colleagues or by mediating communication between healthcare staff and patients (projects 10, 16). Healthcare professionals also provide informal training and support for each other, for example when new staff arrive (projects 3, 8).

As was also noted above, managers enact adaptations to support resilience at both the micro and meso level of the healthcare system. A consideration of the relationships between stakeholders shows that these adaptations are often done in response to needs and demands triggered by other actors in the system. A typical example of this is that managers were found to purposively compose clinical teams and delegate roles and responsibilities on certain shifts in order to capitalise on the experience and competence of available staff members, while optimising care quality for patients or positive working relationships between staff members (projects 1, 3, 6, 9, 10, 14).

## Discussion

The purpose of conducting this stakeholder analysis was to identify and categorise stakeholders relevant to the phenomenon of resilience in healthcare, and to investigate relationships between healthcare stakeholders enacting system resilience. This study, which reasonably reflects its Norwegian setting, has found that patients, family carers, healthcare professionals, managers at both the micro and meso levels of the system, and policy makers emerge as key healthcare system stakeholders involved in performance variability and adaptations that facilitate resilience in healthcare. In this study, stakeholders have been abstracted into groups of broad categories, while acknowledging that there is great variation within these broad groups according to, for example, patient types and characteristics, as well as types of healthcare professionals and the clinical settings they belong to, or the types and function of the diverse stakeholders grouped together as other service providers. There is also variation within the group of managers, for example according to the system level where they operate. The majority of managers identified in this study were from the micro level of the healthcare system, however, we found that managers often have a similar role in facilitating and supporting adaptations regardless of their system level.

Several of the projects included in this study were conducted within hospital inpatient and primary care services for elderly patients. To some extent, these findings reflect those from previous work within the resilient healthcare field [38, 53, 54], which has found that research within the field has largely been focused on empirical settings and actors at the micro level of the healthcare system. For example, Berg and colleagues [38] found that previous resilience research has largely been concerned with how healthcare professionals contribute to the adaptive capacity and resilience of healthcare systems. This has mainly been done by investigating everyday work-as-done and making explicit the adaptations, including problem-solving, communication, sensemaking, and decision-making processes, that healthcare professionals engage in in their day-to-day work.

The key role that healthcare professionals play in the adaptive capacity of the system was clearly indicated in our findings as well, wherein all identified healthcare professionals were categorised as players in the interest/influence grid to indicate that they are the stakeholders that are most likely and able to enact adaptations in everyday clinical practice and thus contribute to healthcare system resilience. The heavy involvement that healthcare professionals have in both performance variation and adaptations is expected since they are the system actors with the most influence and direct control over their own work and decision-making regarding patients and other key healthcare tasks. They also have comparatively high stakes in ensuring that the care they provide to patients is safe and of high quality, which has been noted elsewhere in the literature too [55–58], though healthcare professionals may not necessarily think of their role in maintaining the quality of healthcare in terms of resilience and being a key part of upholding a resilient organisation or system.

In addition to supporting previous research findings on the central role of healthcare staff, this stakeholder analysis adds further knowledge and understanding of the contribution of other key healthcare system actors to resilient healthcare. Notably, this concerns the contribution of family carers and certain groups of patients, as well as the contributions from managers at both the micro and meso levels of the system, and various actors at the macro level including policy makers. Alongside healthcare professionals, family carers emerged as crucial stakeholders in the enactment of adaptations seen in this study. Family carers have significant interest and influence when it comes to supporting and advocating for patients in a range of interactions with other healthcare system actors, though primarily in communication and collaboration with healthcare professionals at the micro level. This is in line with what other studies have found on the importance of family carers’ various contributions to resilient healthcare systems, such as advocating for patients and supporting patients through care transitions [18, 59, 60].

The data show that patients can be directly or indirectly involved as stakeholders in resilient healthcare. In many instances, patients themselves do not have an active role in influencing performance and variability adaptations, rather, they are the subject towards which performance adjustments and adaptations enacted by others are directed. For example, patients often influence performance variability and adaptations in situations where it is their medical condition and related needs that trigger performance adjustments and adaptations in practice. The active players in these adjustments and adaptations directed at patients are healthcare professionals and family carers. However, it is notable that patients’ ability to influence practice adaptations made by other system actors such as healthcare professionals becomes heightened if they have family to support them and advocate on their behalf. Again, this points to the crucial role that family carers have in supporting, coordinating, and advocating for patients throughout their healthcare journeys [61–63].

At the same time, these dynamics between system stakeholders illustrate the uneven power relations that exist within the system and that many patients are likely to have only limited influence on the safety and quality of care because they lack close relationships with other system stakeholders. This was particularly the case for elderly patients who lack the support of relatives. Patients may however have unrealised power which becomes activated through relationships with stakeholders with more influence, typically family. This indicates the importance of less influential stakeholders being able to engage with more dynamic system actors to exert joint influence on care activities and decisions. The key importance of relationship ties and the ability to make use of relationships to achieve shared goals and desired problem-solving within the healthcare context has been discussed in terms of the concept of relational coordination. Relational coordination holds that relationships play a fundamental role in enabling system actors to respond in resilient ways to changes, challenges and pressures, thereby enabling resilient systems [6–9, 64, 65].

The findings from this stakeholder analysis seem to support the assertion that relationships between healthcare system stakeholders are an important aspect of resilience in healthcare and that resilience is supported by social interaction and negotiation, relying on coordination and communication between different actors across both time and physical locations. Relationships between patients and family carers, as well as patient and carer relationships with healthcare professionals appear as particularly relevant to facilitating resilience in healthcare. For example, close, collaborative relationships between healthcare professionals and family stakeholders are important to be able to provide patient centered care adapted to meet the needs of individual patients and their families. In this sense, family stakeholders have an important role and function within the system that cannot be replaced by professional healthcare workers [14]. Future research should explore the contributions of patients and their carers to resilient healthcare systems in more detail.

This stakeholder analysis furthermore found that managers at both the micro and meso levels routinely enact adaptations in response to emerging needs by actively allocating or reallocating staff to certain shifts and by composing shift teams in order to maximise the experience and competence of available staff members. These findings are in line with those described by Fagerdal and colleagues [10] in a study exploring how managers enable adaptive capacity in hospital teams. In that study, managers were found to spend a lot of their time purposively composing shift teams to arrive at the right mix of experience and skills, in order to facilitate the functioning of the teams and to reduce risk to patients. This stakeholder analysis also found that managers actively work to set the scene for adaptations to be engaged in by other stakeholders, for example by providing training opportunities and reflexive spaces for their staff members [66]. This reflects findings from several other studies that have found that one of the crucial ways that healthcare managers engage in quality work and adaptations necessary to uphold overall system resilience is by facilitating competence development and continuous education and training opportunities among staff [10, 67–69].

Adaptations were found to take place at both the individual level (micro) and at the organisational level (meso), often as a response to policy demands and guidelines stemming from healthcare system actors at the macro level. Policy makers at the macro level of the healthcare system are thus important indirect stakeholders in performance variability and adaptations. In this study, they were mainly found to be present through the various policy documents, national guidelines, etc., that they produce, which set the context and parameters of everyday clinical practice. Other system stakeholders, notably healthcare professionals and managers, adapt their work around these policies and guidelines, either by trying to accommodate them to uphold high standards of healthcare quality, or by trying to uphold quality and safety in practice despite what policy documents and clinical guidelines prescribe. This type of adaptation was particularly notable within the mental healthcare setting where healthcare staff routinely adopt an interpersonal, flexible approach to protect and care for patients, thereby necessitating constant trade-offs between emergent, highly context-dependent patient needs and conflicting clinical guidelines [70, 71].

What this stakeholder analysis study adds to the resilience in healthcare literature, therefore, is data concerning stakeholders from various macro level settings of the healthcare system. Empirical studies on how different macro level actors contribute to adaptive capacity and resilient performance in the wider healthcare system have traditionally been lacking [72]. Recent studies have however begun investigating the important influence of stakeholders such as healthcare regulators, external inspectors, and other governmental representatives on the resilience of our healthcare systems [73], with findings suggesting that macro, meso and micro level actors should be considered collaborative partners in efforts to facilitate system-wide adaptive capacity [74]. Our study corresponds with this work by further indicating the need to increase our understanding of the decision-making power and influence that macro level actors exert on system-wide resilience through, for example, the mechanisms and structures they provide [75], and how actors at the micro and meso levels of the system may come to influence macro level stakeholders and the decisions they make regarding healthcare policy and regulation. Furthermore, there is a need to broaden our understanding of what may be regarded as valued adaptations and successful outcomes of resilient performance for whom and when; that is, by which stakeholders and in what context [76–79].

There are some managerial and policy implications of this study. Healthcare professionals and managers alike need to be able to consider the key role of stakeholders such as patients and their family carers in healthcare resilience, and this knowledge must be integrated into practice in formalised ways [80]. Managers therefore have a particularly important role in facilitating a culture of awareness and understanding among their staff of how patients and families contribute to the quality, safety and resilience of healthcare. One way of doing so is by adopting collaborative learning approaches and establishing reflexive spaces for staff where system complexity is acknowledged [81]. Regulators and policy makers for their part must acknowledge their central role as context setters for resilient healthcare in practice. They should be encouraged to have more direct contact and interaction with all the other stakeholders in the healthcare system and integrate multiple stakeholder perspectives into their work, so that all healthcare stakeholders together can create safer, more resilient systems. Furthermore, policy makers together with politicians need to create policy and legislation that acknowledges and supports patients’ and families’ role in the enactment of resilient healthcare services, while providing the funding needed to strengthen this co-production in practice and thus facilitate more collaborative and patient-centred healthcare services [80].

## Strengths and limitations

This stakeholder analysis has been conducted using a sample of empirical projects from the Norwegian healthcare context only and may therefore not provide a full picture of stakeholders relevant to resilience in healthcare in other national settings or cultural contexts. A more comprehensive empirical sample from a more diverse range of healthcare systems and countries may have identified other stakeholders of relevance, such as actors from the pharmaceutical industry, or given a different view of stakeholders’ respective importance in facilitating healthcare system resilience. However, the data material did include a variety of Norwegian healthcare settings, which is a strength of this study and the reliability of its results. The use of different analytical approaches, such as interest-influence and actor-linkage matrices, in this study has provided an in-depth understanding of patients’ and other healthcare system stakeholders’ contributions to resilience. However, application of other stakeholder analysis approaches or other research methods could allow for exploration of further similarities and distinctions in the various stakeholders’ contributions and roles, such as contextual nuances in how managers at different system levels facilitate and support adaptations.

Overall, more research is needed from a variety of settings and perspectives, and using a range of methods for stakeholder analysis, to investigate the characteristics and roles of the key stakeholders relevant to facilitating resilience in healthcare more broadly. It is also important to acknowledge that stakeholder analysis is an inherently iterative process [28]. Consequently, the identification, differentiation and categorisation of stakeholders, including their level of importance, interest and influence regarding a given phenomenon may fluctuate and change over time depending on the nature, needs, and demands of the actors and the circumstances involved [82, 83]. This means that the consideration and engagement of stakeholders involved in healthcare system resilience is a process of continual attunement to the context facilitating or constraining performance variability and adaptive capacity in a given healthcare system. Future research should therefore aim to assess and further differentiate stakeholders’ degree of impact on healthcare resilience relative to multiple and differing contextual factors.

## Conclusion

This stakeholder analysis found that patients, family carers, healthcare professionals, managers, policy makers and regulatory investigators are all in various ways key stakeholders in the enactment of adaptations in a range of healthcare settings and contexts, thus contributing to healthcare system resilience. This study has shown that family carers and healthcare professionals are likely to be the most active stakeholders in the enactment of adaptations, whereas patients contribute both directly and indirectly to the enactment of adaptations. Managers, policy makers and regulatory investigators are likely to be involved by providing the context / setting the scene for the adaptations taking place. Relationships between most stakeholder groups are largely characterised by communication and coordination, and to a certain extent also more formal collaborations, where actors work together to achieve common goals. The most closely linked stakeholder groups, which work together as a team that mutually plan and share resources to accomplish common goals, are patients and family carers; healthcare professionals working with other healthcare professionals; as well as healthcare professionals and their ward/unit managers.

Based on these findings, we would like to make some recommendations for future practice and research, thus going beyond the scope of most published stakeholder analyses [46]. We recommend that healthcare service providers including healthcare professionals and ward/unit managers, as well as other influential stakeholders such as organisational managers, policy makers and regulatory investigators, acknowledge the crucial role that patients and their families may play within everyday quality and safety work and that their contributions to the quality and safety of clinical care must be considered as a vital part of the full picture of what makes healthcare systems resilient. Further research is needed that explores in more depth how patient and stakeholder involvement in resilient healthcare can be developed and supported across all levels of the healthcare system.

### Supplementary Information

**Additional file 1.** 

## Data Availability

The Resilience in Healthcare datasets are available from the corresponding author on reasonable request.

## Abbreviations

SHARE: Centre for Resilience in Healthcare
RiH: Resilience in Healthcare
SNA: Social network analysis
