# Supplementary material for: Identifying, categorising, and mapping actors involved in resilience in healthcare: a qualitative stakeholder analysis
Source: BMC Health Serv Res. 2024 Feb 22;24:230. doi: 10.1186/s12913-024-10654-4 (PMC10882781; doi:10.1186/s12913-024-10654-4)
Supplement: Supplementary file 1 — Additional file 1. [file 12913_2024_10654_MOESM1_ESM.pdf]

Interview guide for individual interviews in RiH project

(English version)

Start the interview with a review of the information letter and signing of the consent form (see separate document).

Information about the content of the interview

In this interview, the purpose is to learn as much as possible from the project you are participating or have participated in, regardless of what phase it is in. We want to understand the background and content of the project in order to form the best possible basis for assessing what the project can teach us about resilience. We will first ask some short questions regarding project details, then some overall and general questions, before going in-depth on certain specific topics. The interview will have a duration of 1-2 hours depending on the project phase (newly started, midway through, completed).

**Project details**	
Title, duration	
Project type (PhD, post-doctoral, larger research project)	
Project phase (newly started, midway through, completed)	
Thematic area (resilience, learning, user involvement, improvement, variability)	
Empirical field (acute care, psychiatric care, hospital, homecare)	
Project aim	
Design, methods, participants, data material	
Collaborators, stakeholders, patient groups, etc.	
Involvement (methods, activities, number, extent)	
Challenges or changes encountered during the project	
Use or development of teaching or learning resources	
Use or development of interventions (improvement project)	
Number of researchers involved, researchers' background	
Other project information	

General questions

Initially, the questions deal with a number of general features of the research project you are involved in. We will go into more detail on several of the areas later, so you will have the opportunity to elaborate and reflect along the way.

1. Which system level(s) (micro, meso, macro) would you say the project covers?

2. Which interfaces between and within organizations are covered in the project?

3. Which patient groups and/or interest groups are central to the project? Which actors are involved at the various system levels?

4. Which (work) practices will the project study/has the project studied?

5. Can you give examples of important changes, areas for improvement, or variations that the actors in the project deal with as part of their work practices? (E.g., new technology, new patients, time pressures, stakeholders, regulations, etc.)

6. Can you give us examples of important contextual conditions (e.g., managerial, regulatory, cultural, financial, work pressures, etc.) that are important for the (work) practices studied in the project? How are these connected to different levels (micro, meso, macro) of the system?

7. What are different collaborative arenas or learning arenas that exist in the field of (work) practice that the project studies? (E.g., meetings, teamwork, simulation, discussion of adverse events, user panels)

8. What theoretical foundation(s) does the project have?

Empirical reflections

These questions are mainly aimed at the projects that have started or completed empirical data collection. If your project is in the start-up phase, you can discuss whether the project *plans to* study the topics below and how this potentially may become visible in the empirical material from the study setting in question. We may also interview you later in the project regarding empirical results.

9. Are there examples of resilience (adaptive capacity, handling of changes and variation, improvement of practice) in the project's empirical material? Can you give one or more examples? Who is involved? What happens in these situations?

10. Are there examples in the empirical material where the actors take measures that enable them to anticipate and be prepared for the various types of changes they have to deal with? Which measures and what do they entail?

11. Are there examples in the empirical material where the actors map and "monitor" quality? What is done, how and by whom? Is "quality data" used as a foundation?

12. Are there examples in the empirical material of whether actors have "room to maneuver" (e.g., enacting quick responses, involving others, bypassing procedures) in order to make decisions and handle changes and variation in (work) practice? What is done, how and by whom? What happens in these situations?

13. Are there examples in the empirical material of how the actors relate to standard procedures? If so, what types of procedures? What happens if the procedures do not work in practice and there is a need for change or adaptation? (E.g., formal decision-making arenas, colleague discussion, "workarounds".)

14. Is it possible to identify barriers and facilitators for resilience in the project's empirical material? Can you give examples?

15. Are there examples in the empirical material where the actors try to influence contextual factors that are important for (work) practice? (E.g., lobbying, changing regulations, better budget frameworks.)

16. Do you look at / plan to look at the handling of serious incidents and/or the application of formal systems and/or informal practices for reporting and learning from incidents within the field you are studying?

17. Are there examples in the empirical material of how serious incidents are handled and how feedback and learning are practiced in that regard? (E.g., reporting, responsibility, individual, system, use of data.)

Theoretical reflections

18. What would you say is the main theoretical contribution of the project?

19. How is the concept of resilience understood/used in the project? (Is a specific definition used? Which elements are included?)

20. How is the concept of quality understood/used in the project? (Is a specific definition used? Which dimensions are included?)

21. How has the theoretical foundation of the project developed over time? (What changes have been made? What new contributions have been added along the way?)

<u>If the project does not use resilience theory or resilience concepts</u>:

22. How would you describe the connection between the theoretical grounding of your project and the field of resilience, as you understand it?

23. Can you say something about how the results of your project potentially may contribute to the theoretical development or understanding of resilience in healthcare?

Questions about patients, families and other stakeholder groups

If the project is in the start-up phase, you can discuss whether it plans to study the topics below and how this potentially may become visible in the empirical material from the study setting in question.

24. Does the project make visible how patients, families, or representatives from other stakeholder groups contribute to resilience in healthcare (adaptive capacity, handling changes and variation, improvement of practice)? Can you give one or more examples? What roles do the stakeholders have? Individual or collective? What happens in these situations?

25. Are there examples in the empirical material of unexpected stakeholder groups or actors playing an important role for resilience in the relevant study setting? Individual or collective?

26. Are there examples in the empirical material where patients, families, or other stakeholder representatives experience their contributions to resilient healthcare as demanding (tasks, responsibilities, dual roles)?

27. Does the project make visible how patients, families, or other stakeholder representatives contribute to quality of care? Which quality dimensions are covered?

28. Does the project make visible how patients, families, or other stakeholder representatives contribute information and knowledge that enables healthcare professionals to prevent or deal with quality challenges (e.g., involvement in investigations, strategy work, etc.)? Can you give one or more examples? What roles do the stakeholders have? Individual or collective? What happens in these situations?

Questions about collaboration and learning

If the project is in the start-up phase, you can discuss whether it plans to study the topics below and how this potentially may become visible in the empirical material from the study setting in question.

29. Does the project make visible how different collaborative or learning arenas work in practice? Can you give one or more examples? Which activities are included? Who participates, facilitates, or evaluates the arenas? Why do they work (e.g., context, participants)?

30. Are there examples in the empirical material of collaborative or learning arenas established at the interface between different organizations (e.g., supervision, patient representatives, municipalities, healthcare providers, user organisations, etc.)?

31. Are there examples in the empirical material of learning from positive events or experiences (e.g., simulation, “positive deviations”, etc.)?

32. Are there examples in the empirical material of how collaborative or learning arenas contribute to resilience (adaptive capacity, handling of changes and variation, improvement of practice)?

33. Does the project make visible how teamwork functions in practice? Can you give one or more examples? (Team composition, factors affecting teamwork?) What happens in these situations?

34. Are there examples in the empirical material of how teamwork contributes to resilience (adaptive capacity, handling changes and variation, improvement of practice)?

35. Does the project develop, implement, and/or evaluate learning resources (e.g., educational modules, simulations, training programs, meeting platforms, webinars, apps, e-dialog forums)? Are these resources used as improvement measures? Which activities are included? Who participates, facilitates, evaluates the learning resources? Why do they work (e.g., context, participants, technology)?

36. Are there examples in the empirical material of how learning resources contribute to resilience (adaptive capacity, handling changes and variation, improvement of practice)?

37. If the project has not used learning resources, do you have any thoughts on how such measures can facilitate collaborative learning in the relevant study setting? (E.g., content, participants, technology.)
